# Effect of voluntary breathing exercises on stable coronary artery disease in heart rate variability and rate-pressure product: a study protocol for a single-blind, prospective, randomized controlled trial

**DOI:** 10.1186/s13063-020-04402-2

**Published:** 2020-07-01

**Authors:** Qing Wu, Lin Liu, Xin Jiang, Yao-Yao Hu, Qiu-Shi Liang, Zhi-Song He, Yuan Xue, Wei Zhu, Zai-Xiang Tang, Yun-Ying Hou, Qi Zhao, Xiao-Hua Wang

**Affiliations:** 1 Department of Cardiology, The First Affiliated Hospital of Soochow University, Suzhou, China; 2 Nursing Department, Wuxi People’s Hospital, Wuxi, China; 3 School of nursing, Soochow University, Suzhou, China; 4 Electrocardiographic room, The First Affiliated Hospital of Soochow University, Suzhou, China; 5 School of Public Health, Soochow University, Suzhou, China; 6 Department of Radiotherapy, The First Affiliated Hospital of Soochow University, Suzhou, China

**Keywords:** Breathing, Stable coronary artery disease, Heart rate variability, Blood pressure, Myocardial oxygen consumption

## Abstract

**Background:**

At present, China has more than 11 million patients with stable coronary heart disease and this is becoming a major public health problem. The pathological changes of coronary heart disease can lead to dysfunction of the cardiac autonomic nervous system, which increases the risk of complications such as malignant arrhythmia (ventricular flutter, ventricular fibrillation, etc.), heart rate, systolic blood pressure, and rate-pressure product (RPP), which is highly correlated with myocardial oxygen consumption and indirectly reflects myocardial blood supply and oxygen consumption. Although the guidelines recommend that such patients take drugs to reduce heart rate and myocardial oxygen consumption, the clinical control of heart rate is still not ideal. Thus, in this trial, we will use voluntary breathing exercises as the strategy of exercise rehabilitation for patients with stable coronary artery disease (SCAD), in order to increase the vagus nerve activity and/or reduce the sympathetic nervous activity, help maintain or rebuild the balance of plant nerve system, improve the time-domain index of heart rate variability, reduce the burden on the heart, and relieve patients’ anxiety and other negative emotions.

**Methods:**

This is a 6-month single-blind, randomized controlled clinical trial that will be conducted in the First Affiliated Hospital of Soochow University. A total of 140 patients who fill out the Informed Consent Form are registered and randomized 1:1 into the Voluntary Breathing Exercises (VBE)-based clinical trial monitoring group (n = 70) or the Routine follow-up group (n = 70). The VBE-based clinical trial monitoring group is given VBE training on the basis of conventional treatment and health education, while the control group received conventional health education and follow-up. The primary outcomes will be measured heart rate variability and RPP. Secondary outcomes will include changes in Self-rating Anxiety Scale, total cholesterol, triglyceride, high-density lipoprotein, low-density lipoprotein, weight, and body mass index.

**Discussion:**

This trial will carry out scientific respiratory exercise for patients with SCAD, which belongs to the category of active secondary prevention for patients, and changes from remedial to pre-protective. VBE is easy to operate and is not limited by time and place. It is important and meaningful to carry out VBE for patients with SCAD. This study will provide considerable evidence for further large-scale trials and alternative strategies for the rehabilitation nursing of patients with SCAD.

**Trial registration:**

Chinese Clinical Trials Registry, 1900024043. Registered on 23 June 2019.

## Background

Stable coronary artery disease (SCAD) is one of the most common types of coronary artery disease. It is characterized by chronic delay and high recurrence. Several studies [1–3] have demonstrated that pathological changes of coronary heart disease can lead to dysfunction of the cardiac autonomic nervous system, which is manifested as hyperactivity of sympathetic nerve and decreased excitability of vagus nerve. Thayer et al. [4] concluded that autonomic nervous dysfunction leads to cardiac dysfunction and cardiovascular disease. Patients are also at an increased risk of complications such as malignant arrhythmia (ventricular flutter, ventricular fibrillation, etc.), increased heart rate, increased systolic blood pressure, and increased rate-pressure product (RPP), which is highly correlated with myocardial oxygen consumption and indirectly reflects myocardial blood supply and oxygen consumption [5], and is earlier than the change of electrocardiography (ECG). RPP is the product of heart rate and systolic blood pressure and has been used as a non-invasive indicator to assess the level of myocardial oxygen consumption since the 1970s. The basis of the common pathophysiology of the risk complications such as angina pectoris and acute coronary events is myocardial ischemia and hypoxia. RPP is closely related to myocardial oxygen consumption (*R* = 0.90) [6], which is not only used to formulate the amount of exercise prescribed, but also the basis for evaluating the symptoms and exercise ability of patients with cardiovascular disease. To some extent, it reflects the tolerance and safety of certain rehabilitation methods of cardiovascular patients. As a consequence, it is of utmost importance to no longer neglect the stability of autonomic nervous function during rehabilitation of SCAD. Heart rate variability (HRV) is recognized as a new non-invasive quantitative evaluation method of autonomic nervous system function developed in recent years [7]. HRV refers to the small difference between cardiac cycles, which reflects the sympathetic and vagus nervous systems regulating the balance state of the cardiovascular system [8]. It was shown to be related to subtle time changes and patterns of each cardiac cycle, while the average heart rate can only appear normal or abnormal. Shen et al. [9] analyzed the results of 24-h dynamic electrocardiogram of 156 patients with coronary heart disease and found that HRV decreased, ejection fraction decreased, and the incidence of cardiovascular events increased.

The research on the effect of respiration on HRV has become a hot topic in recent years [10, 11]. Previous studies have demonstrated that respiration is one of important influencing factors for HRV [12, 13]. Cicek et al. [14] confirmed that deep and slow breathing can make people turn from depression to relaxation and improve the regulation function of human organs or autonomic nerves, so as to improve the adaptability of human body. Voluntary breathing exercises (VBE) refers to a treatment or method of rehabilitation for adjusting breathing behaviors by trainees according to certain breathing patterns (frequency, depth, ratio of expiratory/inspiratory time, chest/belly style) without the use of auxiliary equipment or equipment [15]. Initially, VBE was applied in the prevention and treatment of chronic obstructive pulmonary disease [16], chest surgery [17], and other diseases related to respiratory system. Subsequently, it gradually spread to the treatment of other chronic diseases such as the endocrine and nervous systems [18, 19]. Some authors consider that VBE effectively improved quality of life, reducing symptoms associated with a variety of anxiety and depression [20, 21]. VBE is also applied to treat cardiovascular complaints, such as primary hypertension [11, 16], heart failure [19, 22, 23], arrhythmia [24], and so on. Kawecka-Jaszcz et al. [23] found that respiratory exercise combined with exercise rehabilitation for a period of 10–12 week in patients with chronic heart failure had a better effect on ejection fraction and 6-min walk test, but this study failed to observe the functional changes of autonomic nervous function. Westerdahl et al. [25] showed that respiratory exercise for 2 weeks reduced systolic blood pressure in patients with coronary heart disease, but had no effect on heart rate and diastolic blood pressure, which may be closely related to the short intervention time. A study on the efficacy of VBE by Huang et al. [26] also reported significant reductions in heart rate and systolic blood pressure, and there were no effects on diastolic blood pressure. The meta-analysis of results of independent breathing exercise conducted by the research team in the previous stage proved this [27] (six randomized controlled trial [RCT] articles, all in English), VBE had significant effects on improving resting heart rate, systolic blood pressure, and diastolic blood pressure in patients with coronary heart disease.

However, more research is necessary regarding the effect of VBE on SCAD. In 2014, the American College of Cardiology (ACC) and the American Heart Association (AHA) [28] pointed out that drug therapy and lifestyle intervention are key to controlling or delaying the stable progression of coronary heart disease, reducing complications, disability rate, and mortality rate. As a lifestyle intervention, VBE has become a new way of comprehensive intervention for coronary heart disease. To date, there are no definite data on the effect of VBE in patients with SCAD, particularly in the Chinese population. Thus, we present the protocol of a single-blind RCT to investigate the effects of VBE on SCAD. We hypothesize that VBE is effective for the management of secondary prevention of coronary heart disease.

### Research hypothesis

**Fig. 1:**
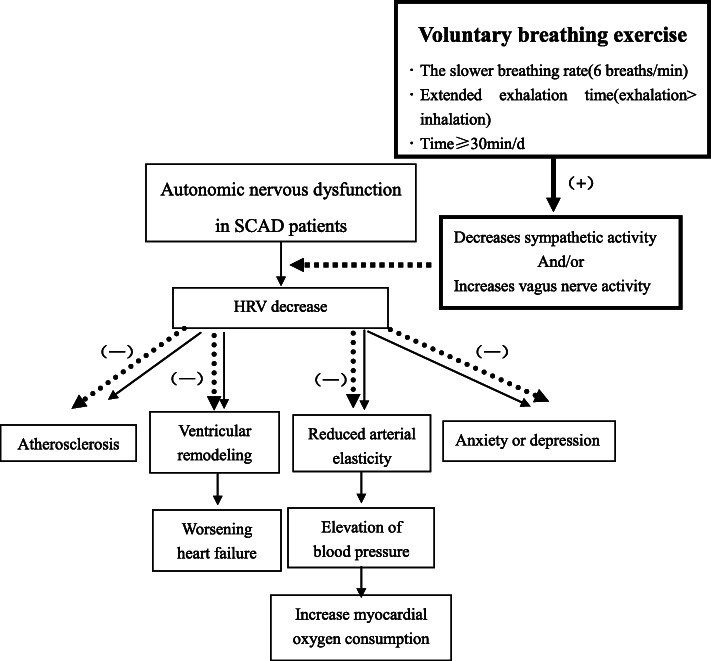
Schematic diagram of research hypothesis

On the basis of routine rehabilitation guidance, in this study VBE will act as an aid in the strategy of exercise rehabilitation in patients with SCAD, in order to increase activity in the vagus nerve and/or reduce sympathetic nervous activity, help maintain or rebuild the balance of the central nervous system, improve the time-domain index of HRV in patients with SCAD, reduce the burden on the heart, and relieve patients’ anxiety and other negative emotions (Fig. 1).

### Objectives

The main goal of this RCT is to evaluate the effect of VBE on HRV and myocardial oxygen consumption in patients with SCAD. First, HRV will be measured using the generally recommended time-domain analysis in 24 h; second, since RPP are highly correlated with myocardial oxygen consumption, it will be used as an indicator to estimate oxygen consumption of the myocardium.

The second objective is to explore the effect of VBE on reducing anxiety in patients with SCAD. Anxiety will be measured using the Self-rating Anxiety Scale (SAS) developed by Zung [29] in 1971.

## Methods

### Study design and setting

**Fig. 2:**
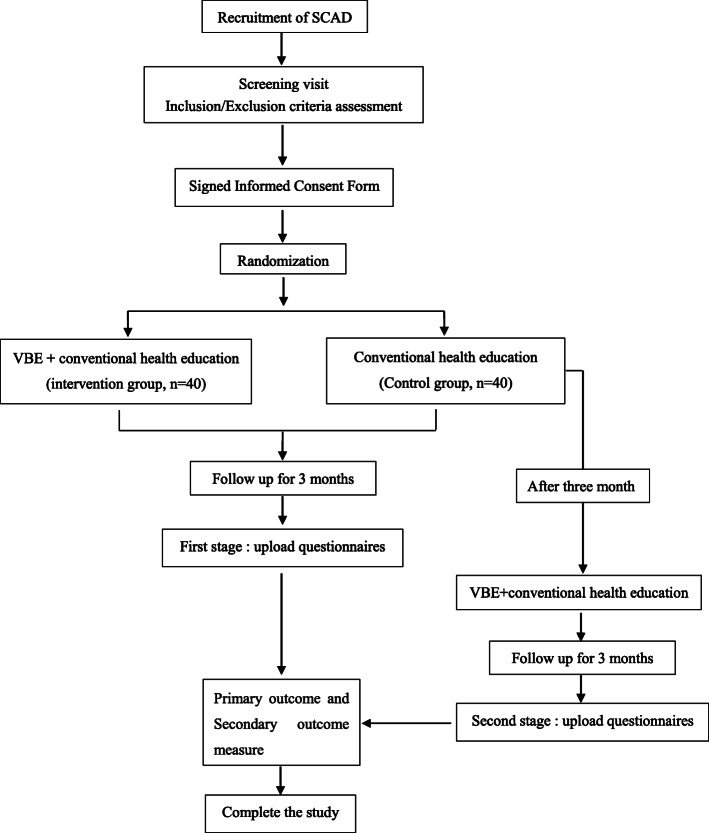
Technology roadmap

This is a 6-month, single-blind RCT that will be conducted in the First Affiliated Hospital of Soochow University. Written informed consent will be obtained from each individual by the researcher after the individual has received a sufficient explanation and period of time in which to make a thoughtful decision. All the patients will be randomized 1:1 into the VBE-based clinical trial monitoring group (n = 70) or the Routine follow-up group (n = 70). The VBE-based clinical trial monitoring group is given VBE training on the basis of conventional treatment and health education, while the control group received conventional health education and follow-up. The two groups will be followed up for 3 months (Step 1). Considering the ethics and the fairness of the resources, we refer to the step-wedge design [30] and give the control group the same VBE guidance and follow up for another 3 months (Step 2) (see the flow diagram in Fig. 2). The Cardiovascular Medicine Department of the First Affiliated Hospital of Soochow University is currently the largest cardiovascular disease diagnosis and treatment center in South Jiangsu Province, China. The department carried out the first PTCA surgery in China in 1983. It has 180 beds, including 20 fully equipped Coronary Care Unit (CCU) beds, four cardiac catheterization rooms with advanced equipment, as well as a cardiac supersonic room and cardiac function room, and 1500 m^2^ cardiovascular laboratory. Annually, an average of 100,000 outpatients, 5000 open-heart surgery candidates, and 400 emergency operations are treated in the department.

### Sample size

The sample size estimation was carried out using G Power 3.1.9.2 software of two population means formulae [31]. We hypothesized that the VBE-based clinical trial monitoring group would be superior to the control group in terms of HRV and myocardial oxygen consumption; the effect rates were calculated with a 0.447 effect size at a 5% significance level and a power of 0.8. This means a sample size of 118 individuals per group was required. With an estimated 20% dropout, the final sample size of 140 individuals was determined (70 in each group).

### Participants

#### Inclusion criteria

Patients will have to meet the following eligibility criteria to be included in the study:

1. Age 45–75 years
2. Meeting the diagnostic criteria of SCAD formulated by by the interventional cardiology group of the cardiovascular disease branch of the Chinese Medical Association, the Atherosclerosis and Coronary Heart Disease Group, and the Professional Committee for the Prevention and Treatment of Thrombosis of the cardiovascular physicians branch of the Chinese Medical Association [32]
3. Heart rate > 60 beats/min [33]
4. Living in Suzhou for at least 6 months from the start of the trial
5. Those who are sane, rational, and able to communicate verbally
6. Those who agree to participate and provide written informed consent

#### Exclusion criteria

Patients will be excluded from the study if:

1. Revascularization has been carried out or conducted in follow-up
2. Combined arrhythmia shows on an ECG
3. Any self-reported respiratory disorder
4. Severe liver and kidney diseases
5. Diagnosed with hypothyroidism or currently taking thyroid medications
6. Diagnosed with moderate or below anemia (hemoglobin [Hb] level < 9 g/dL)
7. With any mental disorders (for example, major depressive disorder, anxiety disorder, substance use, etc.) in the past 12 months
8. Pregnancy and/or practicing yoga/meditation/relaxation/mindfulness on a regular basis
9. Other reasons determined by the investigators that make participation in the clinical trial inappropriate

#### Withdrawal criteria

Participants who meet the criteria summarized below are withdrawn from the study. The individuals who are withdrawn after randomization will be followed up for outcomes. Reasons for withdrawal will be documented in follow-up records and data will be analyzed using the intention-to-treat (ITT) principle [34]:

1. Failed to exercise for 2 weeks in a row
2. Failed to have three consecutive telephone connections during the follow-up period
3. Patients who quit for various reasons

### Randomization

Eligible participants who will provide written consent to participate in this clinical trial will be randomly assigned to a treatment group or a control group with an allocation ratio of 1:1. Random sequencing will be generated by an independent professional statistician using the EXCEL to generate 140 random numbers. The serial numbers assigned to each patient are kept in an opaque, sealed envelope. All the participants will be asked to pick one sealed envelope and pass it onto the research study team member. The participants will be randomly assigned to two groups. Each group will have 70 individuals.

### Blinding

The research study team member who enters information about a participant’s eligibility is the only person to know the participants’ allocation until the participant has completed the entire protocol. The research team member involved in the subjective efficacy assessments is blinded to the participant’s group allocation to receive VBE or conventional treatment. Participants are similarly blinded to their own group allocation and they will be cautioned not to remark on the VBE exercises that they receive to the research study team member performing the outcome measures. Results from the outcome measures will not be revealed to the participants until after all recruitment, treatment, and assessments have been performed for all 140 participants.

### Procedures

#### Study schedule

The items to be measured at each visit are presented in Table 1.

**Table 1:** Schedule of enrollment, intervention, and assessment

	Screening Visit 1 (day −5 to − 2)	Post allocation						Close-out
		Visit 2 (day 0)	Visit 3 (week 1)	Visit 4 (week 2)	Visit 5 (week 3)	Visit 6 (week 4)	Visit 7 (week 8)	Visit 8 (week 12)
Informed consent	X							
Inclusion/exclusion criteria	X							
Randomization		X						
Intervention		X	X	X	X	X	X	X
Vital signs	X	X	X	X	X	X	X	X
Body measurement^a^	X	X	X	X	X	X	X	X
Basic information^b^	X							
Heart function classification	X							
Medical history^c^	X							
General physical examination	X							X
HRV	X							X
RPP	X			X			X	X
Self-rating Anxiety Scale	X							X
Laboratory tests^d^	X			X				X
Heart Doppler ultrasound indexes^e^	X							
Compliance monitoring				X				
Adverse event monitoring			X	X	X	X	X	X

^a^ Height and weight, but only weight for visit 2 and follow-up

^b^ Age job, education level, dietary habit, smoking habits, drinking habits, etc.

^c^ Including general medical history and family history of coronary heart disease

^d^ Including triglyceride, total cholesterol, high-density lipoprotein, low-density lipoprotein, and fasting plasma glucose

^e^ Including left ventricular ejection fraction, left atrium dimension, left ventricular end-diastolic dimension, left ventricular end-diastolic volume, left ventricular end-systolic volume, etc.

#### Baseline assessment

After the screening visit, if a participant fulfills the inclusion criteria and has signed the informed consent form, he will be assigned to the baseline assessment. The baseline assessment takes place at visit 1. Baseline assessment includes patients’ basic information (such as age, gender, marital status, occupation, level of education, dietary habits, income, family history, co-morbidities, number of diseased coronary vessels, previous myocardial infarction, history of stent implantation, etc.), heart function classification, physical anthropometry indexes (height, body weight, blood pressure, heart rate, BMI, RPP, and HRV), heart Doppler ultrasound indexes (left ventricular ejection fraction, left atrium dimension, left ventricular end-diastolic dimension, left ventricular end-diastolic volume, left ventricular end-systolic volume, etc.), testing items (triglyceride [TG], total cholesterol [TC], HDL-C, LDL-C, and fasting plasma glucose), questionnaires (SAS).

#### Intervention group

After enrollment, the intervention group was given the following measures on the basis of receiving the routine health education. Each patient in the intervention group will be assigned a health management document and have a follow-up timetable.

1. Development and distribution of manuals: the Chinese Biomedical Literature Database (CBM) will be searched using the Chinese search terms “health management,” “diet,” “exercise,” “heart rate,” and “blood pressure” and the Cochrane Library, JBI, and Medline will be searched using the corresponding English search terms. After two rounds of expert consultation, the health education manual for patients with SCAD will be constructed and distributed to each patient in the intervention group upon discharge.
2. One-to-one telephone follow-up or home visit: the patient will be followed up after discharge. Follow-up time is 3 months for a total of 12 weeks (once a week in the first month and once a month after that until 3 months). Two follow-up methods will be adopted: telephone and home visit. The telephone follow-ups last about 5–10 min each. Holding time could be extended if there is any problem consultation. Home visits will be scheduled with patients in advance and will last about 30–60 min each. During the follow-up, we will address the patient’s doubts and evaluate the effectiveness of the intervention.
3. VBE guidance: before VBE, patients should rest for 5 min and then do it in a well-ventilated room with appropriate temperature. The researchers help patients take comfortable positions (supine, repose, and standing are all acceptable, sitting is recommended), straighten their head and back, and relax their whole body. The researchers instruct patients to extend the duration of exhalation and inhalation, and to minimize the breathing rate to 6 breaths/min. Participants practice silently counting from 1001 to 1004 on the inhale phase and 1001–1006 on the exhale phase to control the breathing rhythm and slow down the frequency. Participants are also encouraged to use abdominal breathing (a bulge in the abdomen when inhaling and a depression when exhaling) to deepen breathing depth and reduce breathing rate. Practice time: the total time per day is more than 30 min [25], which can be delivered in batches, but for at least 10 min at a time and at least five times a week [12].

Attention: The participants should breathe in and out of the nose during the whole process. The intensity of the exercise should not cause breath-holding or any physical discomfort. If discomfort occurs, participants may rest for a while and continue the exercise if no discomfort is present. For the first 3 days of the study, participants are asked to complete exercises in an outpatient classroom and the researchers will assess whether the exercises are correct or if they need to correct wrong breathing patterns.

4. Exercise log: each participant is given an exercise log and asked to record the number and time of exercise every day. These data can be used to assess intervention compliance.
5. Network support: the research group will make self-breathing exercise material or video and transmit it to each participant through the network, so that participants could browse at any time.

#### Control group

1. Routine education: on the first day after enrollment, researchers explain relevant knowledge of SCAD to patients, including risk factors, diet, weight control, physical exercise, blood pressure, lipid management, etc.
2. Telephone follow-up: once per month for 3 months, each call lasting about 5–10 min, receiving telephone counseling from patients.
3. Post-intervention: the control group will be given the same intervention measures 3 months later, and the effects of the control group will be compared before and after the intervention.

### Outcome

#### Primary outcome measure

The change in the HRV and RPP will be used as the primary outcome measure. The mean difference between the change scores of the two groups will be calculated.

1. HRV: currently, a 24-h time-domain analysis index is generally recommended for HRV detection [35]. All patients will be tested by 12-channel dynamic ECG (TLC5000) to detect the time-domain index of HRV between visit 1 (baseline) and visit 8 (after treatment). During the examination, participants are required to have adequate sleep overnight, maintain a quiet and peaceful environment, avoid extremely intense exercise, avoid changes in posture and mood swings, avoid drinking stimulating drinks such as coffee, and try not to take drugs that may interfere with autonomic nerves.

Indicators include: (1) standard deviation of normal-to-normal (SDNN), SDNN is the SD of 24 h continuous RR interval; (2) SD of average 5-min normal-to-normal intervals (SDANN), SDANN is the SD of the mean of continuous RR intervals every 5 min for 24 h; (3) square root of the mean squared differences of successive normal-to-normal intervals (RMSSD), RMSSD is the root mean square of all RR intervals within 24 h; (4) percent of the number whose difference between adjacent normal-to-normal intervals are > 50 ms (PNN50), PNN50 is the percentage of sinus beats in which the difference between the two adjacent normal RR stages was > 50 ms at 24 h; (5) mean of SDs of normal-to-normal intervals for each 5-min period (SDNNindex), SDNNindex is the mean of the SDs for 5-min segments.

All dynamic electrocardiograms are completed by professionals in the electrocardiogram room of the same hospital; HRV time-domain indexes are automatically analyzed and calculated by the electronic computer.

2. RPP: RPP is used to estimate myocardial oxygen consumption [36]. The normal value of RPP is < 12,000, and the smaller the product, the more stable the patient’s condition. In this study, an electronic blood pressure monitor (OMRON, HEM-4011C) will be used to measure patients’ heart rate and blood pressure at visits 1, 4, 7, and 8. The measurement time is scheduled to be from 14:002 to 15:00 and the room temperature is maintained at about 25 °C in the same outpatient classroom. After sitting for 15 min, the patient will be measured at the right elbow. Patients should ensure that they do not eat or drink strong tea, coffee, or other stimulating drinks 1 h before the measurement.

#### Secondary outcome measure

1. SAS: the Zung SAS is an anxiety measure designed by William WK Zung in 1971 to quantify the level of anxiety for patients experiencing anxiety-related symptoms [28]. The SAS test is self-administered, with each response using a 4-point scale, ranging from “none of the time” to “most of the time.” There are 20 questions with 15 increasing anxiety level questions and five decreasing anxiety questions. According to SAS standard scores, the respondents are divided into four categories: no symptoms of anxiety (< 50 points); mild anxiety (50–59 points); moderate anxiety (60–69 points); and severe anxiety (≥ 70 points). The scale has been widely used in China and its validity and reliability have been verified.
2. Metabolic profile: TC, TG, HDL-C, and LDL-C.
3. Body composition: weight and BMI.

#### Safety outcome measures

The safety assessment will be performed for the participants who have received VBE training more than once. The individuals’ vital signs and general physical status will be examined at every visit. The occurrence of adverse events (AE) will be checked at visits 3, 4, 5, 6, 7, and 8. The investigators should educate the individuals to report any AE that occurs after training. All AEs that occur after the start of this trial should be documented in the case report form, whether or not they are related to VBE. All AEs will be evaluated for causal relationships.

#### Protocol supplementary

The researchers will collect the exercise logs of the patients after 2 weeks of exercise for the purpose of calculating exercise compliance. The rate of compliance will be calculated as: compliance (%) = [continuous regular exerciser/total intervention group] * 100. Clinical trials will be continued only if compliance is ≥ 70%.

### Data management and quality control of data

Both the electronic medical record and web-based electronic database will be used to manage individual participant data. To protect confidentiality, the files are stored in a secure and locked place and manner. Quality control of the data will be handled at two different levels: the investigators will be required to ensure the accuracy of the data as the first level of control when they input the records in electronic medical record. The second level will include data monitoring and validation that will be carried out by two full-time graduate students who will not be involved in the intervention or data collection. The database will be locked under the orders of the Principal Investigator. All the data will be double-inputted into the computer using Epidata3.0 software. After checking and proofreading, SPSS statistical software (SPSS Inc., Chicago, IL, USA) will be used for data analysis. Nobody can have access to the database without authorization from the Principal Investigator.

### Statistical analysis

The Kolmogorov–Smirnov test and p-p diagram analysis will be used to test the normal distribution of continuous variables. Continuous variables will be presented by mean ± SD if they are normally distributed. Mann–Whitney U test will be used and expressed by median with interquartile range if they are not normally distributed. Chi-square or Fisher’s exact tests will be used for comparison of dichotomous data. Both per-protocol (PP) and ITT analyses will be used to determine the robustness of the evidence. An independent statistics expert will perform the statistical analysis in a blind manner. All statistical analyses of the data will be performed by using SPSS software version 21.0 (SPSS Inc., Chicago, IL, USA). A multivariate analysis will be performed to identify the factors associated with outcome indicators. Statistical significance is obtained with a *P* value < 0.05.

## Discussion

HRV is currently recognized as one of the indicators reflecting impaired autonomic nervous function [37], which is affected by sympathetic and parasympathetic nerves and shows fluctuations. Studies [38] have shown that myocardial ischemia caused by coronary heart disease promotes a long-term high level of sympathetic nerve activity, changes the normal balance between sympathetic and vagus nerves, and shows a decrease in HRV on the dynamic ECG.

Ferrari et al. [39] found that multiple time-domain and frequency-domain indexes of HRV in patients with coronary heart disease were significantly reduced, and autonomic nerve function was impaired to varying degrees. SDNN, HRV’s time-domain index, reflects the overall level of HRV; SDANN mainly reflects sympathetic tension; SDNNindex is affected by vagus tension and variable sensory tension; RMSSD mainly reflects vagus tension; and PNN50 variation trend is basically the same as RMSSD [40]. The high-frequency components of HRV, also known as “respiratory components,” synchronize with respiratory movements and regulate HRV through a central mechanism and mechanical influence. Therefore, in recent years, scholars have focused on the effect of respiration on HRV [41]. Rossi Caruso et al. [42] conducted an intervention on 10 male patients with heart failure and 10 healthy patients, respectively measuring heart rate and RR interval during and after lying down, sitting down, walking, breathing training, and found that deep breathing training could improve HRV in patients with chronic heart failure.

Chinese guidelines from 2018 for the diagnosis and treatment of SCAD [32] pointed out that the heart rate should be controlled at 55–60 times/min during the treatment of SCAD to reduce myocardial oxygen consumption, and recommended that patients with SCAD should use beta blockers as early as possible to slow down the heart rate and reduce myocardial contraction. Thus, heart rate control is one of the most important measures in secondary prevention of coronary heart disease. However, even if such patients take drugs regularly in China, the heart rate still cannot reach the target rate, and only 35.9% patients reach the heart rate standard [43]. Multiple studies have shown that increased heart rate can induce the occurrence of cardiovascular diseases, especially cardiovascular events [44, 45]. Framingham followed up 5209 male patients for 36 years; the results showed that the all-cause mortality of cardiovascular diseases including coronary heart disease gradually increased with the increase of heart rate [46]. The research results of Jae et al. [47] showed that rapid heart rate was likely to cause malignant ventricular arrhythmia, which was related to long-term coronary artery calcification.

VBE includes controlled deep breathing, lip contraction breathing, and abdominal breathing [26]. VBE can effectively interfere with autonomic nerve function and can be reversely adjusted according to the characteristics of autonomic nerve function under different pathological conditions. If the basic state is characterized by excessive excitability of sympathetic nerve, its excitability can be reduced, but if the vagus nerve activity is low, it can increase its activity, etc., so as to have a corresponding impact on blood pressure and heart rate [48]. Deep and slow mode VBE can increase parasympathetic activity, reduce sympathetic activity, and improve cardiovascular function [26].VBE is not limited by time, place, auxiliary equipment, etc., which is one of the methods of lifestyle intervention. VBE is one of the frequently used alternative strategies for rehabilitation exercises in patients with cardiovascular diseases, which can be a new approach for the comprehensive intervention of coronary heart disease [7, 11].

Our trial has several strengths. First, the purpose of this study is to explore the effects of VBE on HRV and RPP in patients with SCAD. There have been studies on the application of VBE in clinical fields at home and abroad; our research team studied the effect of VEB of 1 month on the heart rate and blood pressure of patients after PCI in the early stage, achieving good results. Therefore, we have a solid foundation and stable research direction. Second, the study, through health education guidance and breathing exercise intervention, fully arouses the awareness of self-management of patients with coronary heart disease, explores more practical and effective methods of rehabilitation and exercise for patients, improves patients’ active participation consciousness and enthusiasm of disease treatment, and provides basis for the implementation of rehabilitation and exercise methods for clinical medical staff and patients. Third, this study explores the effect of VBE on HRV of patients with SCAD, which is a secondary prevention of cardiovascular and cerebrovascular diseases, which has not been involved in China.

However, there are a few limitations of this study. First, due to limited funds, the study is conducted only in Suzhou. Second, the implementation of VBE is carried out in each patient’s family. Although self-monitoring records will be issued to each patient, the possibility of random recording may occur, and the researchers cannot objectively quantify the compliance of patients with VBE. In addition, this study cannot avoid the influence of drug adjustment on heart rate and blood pressure during the intervention period. Therefore, we will compare the medication status of the two groups when collecting baseline data.

After completion of this study, we will carry out large-sample, multicenter, and high-quality RCTs in future studies to further verify the role of the implementation of VBE in the maintenance and promotion of health and provide reliable evidence-based evidence for the promotion and application of VBE in clinical practice. This protocol was written according to the SPIRIT 2013 Statement [49]. The SPIRIT Checklist can be found as an additional file (see Additional file 1).

## Trial status

Protocol version number and date: version 1.0, 1 March 2019. The study was registered at Chinese Clinical Trials Registry on 23 June 2019. Recruitment was started on 1 June 2019 and is expected to end in December 2020. So far, 16 patients have been recruited for this trial.

## Supplementary information

**Additional file 1.** SPIRIT 2013 checklist: recommended items to address in a clinical trial protocol and related documents.

## Data Availability

The datasets generated and analyzed during the current study are not publicly available due to technical problem but are available from the corresponding author on reasonable request.

## Abbreviations

ACC: American College of Cardiology
AE: Adverse event
AHA: American Heart Association
BMI: Body mass index
CBM: Chinese Biomedical Literature Database
CCU: Coronary Care Unit
ECG: Electrocardiography
HDL-C: High-density lipoprotein
Hb: Hemoglobin
HRV: Heart rate variability
ITT: Intention-to-treat analysis
LDL-C: Low-density lipoprotein
PNN50: Percent of the number whose difference between adjacent normal-to-normal interval is > 50 ms
PP: Per protocol
RCT: Randomized controlled trial
RMSSD: Square root of the mean squared differences of successive normal-to-normal intervals
RPP: Rate-pressure product
SAS: Self-rating Anxiety Scale
SCAD: Stable coronary artery disease
SDANN: Standard deviation of average 5 min normal-to-normal intervals
SDNN: Standard deviation of normal-to-normal
SDNNindex: Mean of standard deviations of normal-to-normal intervals for each 5-min period
TC: Total cholesterol
TG: Triglyceride
VBE: Voluntary breathing exercises
